# Editorial: Current Knowledge on Pathogenic and Endosymbiotic Tick-Borne Bacteria

**DOI:** 10.3389/fvets.2022.900510

**Published:** 2022-04-25

**Authors:** Mourad Ben Said, Sandra Diaz Sanchez, Armanda Bastos, Cornelia Silaghi

**Affiliations:** ^1^Higher Institute of Biotechnology of Sidi Thabet, University of Manouba, Manouba, Tunisia; ^2^Laboratory of Microbiology at the National School of Veterinary Medicine of Sidi Thabet, University of Manouba, Manouba, Tunisia; ^3^SaBio, Instituto de Investigación en Recursos Cinegéticos (IREC-CSIC-UCLM-JCCM), Ciudad Real, Spain; ^4^Department of Zoology and Entomology, University of Pretoria, Pretoria, South Africa; ^5^Institute of Infectology, Friedrich-Loeffler-Institut, Federal Research Institute for Animal Health, Greifswald, Germany

**Keywords:** ticks, hosts, tick-borne pathogens, tick-borne endosymbionts, vectors, tick-borne diseases

This Research Topic converges different original and review contributions highlighting tick associations with microbial communities. These contributions shed light on the fields of (i) diagnosis, epidemiology, and phylogeny of pathogenic and endosymbiotic tick-borne bacteria, and (ii) tick microbiota exploration.

The first contribution to this topic (Körner et al.) reviewed prevalence studies of *Coxiella burnetii* in 25 tick species collected from 23 European countries. The authors show that in half of these studies, no *Coxiella* DNA was detected and that in most studies, no distinction was made between *C. burnetii* and *Coxiella*-like endosymbionts (CLEs), underscoring the need to develop more discriminative methods to better clarify the role of ticks in the transmission of Q-fever.

For the first time, Beliavskaia et al. reported *in vitro* isolation of the bacterial symbiont *Spiroplasma* from third-generation adult male and female *Ixodes persulcatus* maintained in a laboratory colony for over 4 years. This study confirmed that co-cultivation of internal organs with tick cell lines is a simple and effective technique for *in vitro* isolation of intracellular tick symbionts such as *Spiroplasma* species.

To better understand the epidemiology of *Rickettsia* species in Tunisia, Belkahia, Selmi et al. demonstrated the occurrence of rickettsial bacteria in ticks of the *Rhipicephalus* genus infesting small ruminants in Tunisia. The study confirmed the occurrence of human-pathogenic *Rickettsia* species in *Rh. sanguineus* s.l. and *Rh. turanicus* ticks collected from small ruminants in Tunisia. These findings expand knowledge on ticks collected from domestic animals, and highlight the range of infectious agents that may be transmitted with ticks to humans and animals. The presence of *Rickettsia* in ticks is also investigated by Chitanga et al., where the potential risk of human infection by zoonotic *Rickettsia* species in southern Zambia was addressed.

Continuing with epidemiological studies, El Hamiani Khatat et al. presented a systematic review that summarized the wide epidemiological data published on *Anaplasma phagocytophilum* in canine species and described the clinicopathological aspects of canine granulocytic anaplasmosis that are available in the few case series and reports. In this manuscript, the authors gathered all data on *A. phagocytophilum* in dogs that can be valuable for researchers and identified important information gaps to guide future research.

Contributing to shed light on the epidemic situation of tick-borne pathogens, Wang et al. assessed the distribution and risk factors of *Anaplasma* spp. and *Ehrlichia chaffeensis* in yaks (*Bos grunniens*) and Tibetan sheep (*Ovis aries*) from Qinghai, China. This first report of *A. capra* and *E. chaffeensis* infection in yaks in China emphasized the need to assess the threat posed by these pathogens to veterinary and public health. Within this context, Belkahia, Ben Abdallah et al. revealed a greater diversity of Tunisian *A. marginale* isolates compared to worldwide isolates and strains by using a single gene typing method. In addition, the analysis of the vaccine candidate OmpA protein demonstrated that this antigen appears to be highly conserved, suggesting that the minimal intraspecific modifications will not affect the potential cross-protective capacity of humoral and cell-mediated immune responses against multiple *A. marginale* strains. To highlight the need for surveillance and control programs for transboundary diseases (TBDs), Galay et al. showed the high prevalence of *A. marginale* in Luzon, Philippines, provided the first molecular evidence of *E. minasensis* in the country, and confirmed the presence of *E. minasensis* in naturally-infected cattle and *Rhipicephalus microplus* ticks.

Regarding the role of the tick microbial community in the success of tick-borne pathogens, Aguilar-Díaz et al. contributed with a review addressing various mechanisms occurring at the microbiota-pathogen interface and its contribution to tick fitness, adaptation, and immunity and identifying potential targets for anti-tick vaccine development. The combination of conventional and high-throughput sequencing methods was implemented by Takhampunya et al. to provide important information on bacterial community composition and co-infection rates in questing ticks in Thailand with implications for animal and human health.

A different approach is presented in Cull et al. Indeed, this research provides evidence that the endosymbiont of *Ixodes scapularis, Rickettsia buchneri*, exerts an inhibitory effect on the growth of pathogenic tick-borne bacteria in cell culture and possesses two gene clusters encoding putative antibiotic biosynthesis machinery. This suggests that in addition to being a potential nutritional endosymbiont, *R. buchneri* may also prevent pathogenic *Rickettsia* species from occupying the ovaries that may be detrimental to the tick's biology. Supportive evidence from *in vivo* studies could have important implications for our understanding of rickettsial interference and the vector competence of *I. scapularis* for SFG rickettsiae.

In another context, control of tick-borne pathogens generally depends on three main strategies, namely vector control, vaccine development, and the administration of antimicrobial drugs. For *Babesia* protozoa, one of the promising strategies against species of this genus is to control the receptor-ligand interactions of parasite molecules and their target cells, such as RON-AMA-1. Therefore, in this Research Topic, Li et al. screened 502 compounds from the natural product compounds (NPCs) against the *in vitro B. bovis* growth and against the *in vivo B. microti* growth. Findings obtained by these authors indicate the richness of natural product compounds by new potent anti-babesian candidates, and the identified potent compounds, in particular Narasin, could be used for the treatment of animal babesiosis.

As Editors of this Research Topic, we would like to acknowledge all authors who have contributed high-quality original articles and very interesting reviews. We hope that the reader will find in this Research Topic a useful reference for the state of the art in the knowledge on pathogenic and endosymbiotic tick-borne bacteria on two broad aspects: (i) the epidemiology of pathogenic and endosymbiotic tick-borne bacteria, and (ii) the tick microbiome-pathogen-endosymbiont interface.

## Author Contributions

All authors listed have made a substantial, direct, and intellectual contribution to the work and approved it for publication.

## Conflict of Interest

The authors declare that the research was conducted in the absence of any commercial or financial relationships that could be construed as a potential conflict of interest.

## Publisher's Note

All claims expressed in this article are solely those of the authors and do not necessarily represent those of their affiliated organizations, or those of the publisher, the editors and the reviewers. Any product that may be evaluated in this article, or claim that may be made by its manufacturer, is not guaranteed or endorsed by the publisher.

